# Inflammation, Abscess and Necrosis, Their Cause and Treatment

**Published:** 1879-02

**Authors:** J. M. Porter

**Affiliations:** TOLEDO, OHIO.


### AMERICAN JOURNAL

OF

### DENTAL SCIENCE.

Vol. XII. THIRD SERIES—FEBRUARY, 1879. No. 10.

### ARTICLE I.

### *Inflammation, Abscess and Necrosis: their Cause and Treatment.*

BY. J. M. PORTER, D. D. S., TOLEDO, OHIO.

Delivered before the Dental Class of the University of Michigan, November 18th, 1878.

Gentlemen:—The pathological conditions to which I shall invite your attention are so intimately associated that I can not discuss any one of them without including the others. And although they are hackneyed subjects, they are nevertheless, worthy your attention, both on account of their importance as connected with your daily practice, and because of the fact that their causes and treatment are but indifferently understood by the average dentist.

First, inflammation. This term has been used from a very remote period, to denote collectively, the heat, pain, redness and swelling, which are the four chief characteristics of the pathological conditions, known by the above title. The redness, heat and swelling are physical signs, and these more certainly prove the existence of this peculiar diseased condition, than does pain, which is a symptom depending upon the vital property, sensibility, and is often present when inflammation does not exist. For instance, in neuralgia, gastralgia, perforated intestine, etc. So long as the excessive pain lasts, all the functions suffer; faintness and exhaustion are apt to ensue, and, if no relief comes, the prostration may be fatal. Here, to mitigate or remove the pain, is the first and pressing indication. On the other hand, pain may fail to occur when inflammation is present, depending, as it does, upon the natural sensibility of the parts, the degree in which determination of blood predominates, and the tension or pressure induced. The severest pain arises, when all these circumstances co-operate, as in the inflammation of the pulp of a tooth, the sheath of a nerve, or the lining of a bony canal, like the auditory meatus. In other cases, the pain is chiefly felt when the inflamed part is pressed upon or stretched. Thus, the pain of peritonitis is felt, when the abdomen is compressed, or when its walls are strained, as in coughing; and likewise, the stitch of pleurisy is felt, on taking a full breath. Many other similar examples might be mentioned.

This condition known as inflammation, is often preceded by two other conditions, known as congestion and determination of blood. Congestion is characterized by an excess of blood in the diseased part, with diminished motion of that blood, and the latter feature distinguishes the condition from determination of blood, which is also characterized by excess of blood in the part diseased. But instead of the motion of the blood being diminished, as in congestion, it is increased. Another prominent distinguishing feature in congestion, is the fact that the arteries are not enlarged, as in determination of blood and inflammation. Congestion may be caused in various ways, the most frequent causes being over excitement of the vessels, by any influence which acts as an irritant, such as poisonous gases, or mechanical contact with abnormal substances. These two include the chief causes of congestion, which you will probably be often called upon to treat. The most important measures to be adopted for the removal of congestion, are such as are addressed to the causes of the disorder. Pressure may be used as a remedy. This forms a chief part of the useful operation of bandages, adhesive plasters, and even of poltices, in various external congestions. Friction is a modification of pressure, especially suited to some forms of congestion, being calculated to communicate the motion that is defective, as well as to support the weak vessels. Another dose of remedies for congestion, comprehends such influences as promote the contraction of the dilated vessels, by augmenting their contractility or tone. It is in this way that astringents and cold operate. The utility of astringents in congestion is limited, by the fact that they contract the arteries, more in proportion, than the capillaries and veins, which are the chief parts distended. Hence their action may still further arrest the motion of the blood, and increase the difficulty. Depletion is doubtless the safest and surest remedy for such cases as you will be called upon to treat. The same remedies are applicable to determination of blood ; the difference in treatment being, that cold applications are more likely to produce contraction of the arteries, which are, in this disease, the portion demanding special attention. Inflammation may be said to comprehend both of the other conditions, to which I called your attention, and in addition, is a still further deviation from the natural condition. The pathological definition, already given, to distinguish inflammation from the other conditions, (in one of which we have increased, and in the other diminished motion of the blood,) is further illustrated by the strong pulsation of arteries leading to the inflamed structure, and in the stagnation of much blood in the structure. In addition to the pathological definition, the outward character of inflammation may also be briefly defined by the four signs, which from the time of Celsus has been considered distinctive of its presence, and to which I have already referred, namely—redness, heat, pain and swelling.

These symptoms are, it is true, sometimes produced by congestion, and by determination of blood, but in a degree less marked, and for a shorter time, than in inflammation. And although there are cases and forms of inflammation in which it is not possible to detect all these marks, they may still be said to constitute its most general character. In common with other varieties of local hyperæmia, inflammation owes the production of redness to the excess of blood in the part, and this redness is heightened by a peculiar concentration of the blood corpuscles in the inflamed vessels, and this is also the cause of some of the results of the process. As in determination of blood, the heat and pain are in part due to the increased motion of the blood, but they are exaggerated by the motion being opposed by obstruction. As in other forms of hyperæmia, the swelling arises partly from over-distention of the blood vessels, and partly from effusions from them, but these effusions in inflammation differ from those of congestion and simple determination, for they depart still farther from natural quantity and quality. The local exciting causes of inflammation, comprehend irritants, whether mechanical, chemical or vital. In the practice of dentistry, the first named may be regarded as the chief cause. Poisons also tend to produce local inflammation, and indirect causes are of common occurrence. For instance, such as first produce congestion, which subsequently becomes inflammation. Inflammation is also distinguished from congestion, by the more abundant effusions, the more florid hue of the redness, the stronger beating of the arteries, the augmented quantity of blood flowing from the veins, indicating increased motion of blood instead of diminished.

The foregoing considerations warrant the conclusion, that the process of inflammation is essentially more complex, as well as further removed from the natural state, than either congestion or determination of blood; that it includes, in fact, both of these conditions, together with certain changes in the blood within the vessels, and leads to further results, more extensive and varied than those that follow from congestion and determination. There is increased motion, or determination of blood, to the affected part, with a more or less obstructed flow through it; the force of the increased flow being partly expended on the arterial portions of the obstructed vessels, and partly diverted into the collateral channels so abundantly supplied by the anastomosis of vessels. The obstruction in the vessels in the inflamed part, seems to be due to several circumstances, which vary considerably in different forms and degree of inflammation. Thus in those which begin with congestion, the obstruction may depend much on the increased mass of blood in the distended capillaries, and the impaired elasticity of their coats; and in part on the diminution of the osmotic force which naturally favors the passage of blood through the capillaries. Changes in the blood probably ensue sooner or later in all instances, and tend to increase the obstruction; but it is when the inflammation originates in direct irritation, as from a thorn in the skin, or a bruise, or wound in the flesh, that the blood in the vessels, seems most speedily to become a cause of obstruction, even in opposition to an increased current directed on it, through the enlarged arteries, and of these blood changes, a diminution in the osmotic properties, may be one, the formation of pale adhesive granules and compound cells, is another, possibly the agglomeration of the red corpuscles may be a third; and the result of this co-operation is, that more or fewer of the capillaries are obstructed by a mass of red and white corpuscles, so amalgamated, as to be no longer distinguishable. It is this combination that leads to the changes which characterize inflammation, and which, in extent and variety, exceed the changes from any other kind of hyperæmia. The effusions from inflamed vessels, are, at an early period, much the same as those produced by tense congestion and determination of blood, but they commonly occur in greater abundance, contain more animal, fatty, and saline matter; and, as the inflammation advances, sometimes present appearances not found in cases of mere congestion or determination. Thus the effusion at first is a thin serum, causing swelling in complex textures, accumulating in the dependent parts of serous cavities, or diluting the secretion of the more simple mucous membranes. But soon fibrine is also effused, a part of which may concrete into coagulable lymph, or still remain dissolved, as in the liquor sanguinis. Fatty matter is also present, both in the liquor sanguinis, and in coagulated fibrine. Thus an inflamed pleura becomes coated with film of lymph, and the clear body effused into the sac when removed from the body sometimes spontaneously separates into a fibrinous clot and serum. In mucous membranes, there may be a thickening of the submucous texture, and the mucous secretion often becomes unusually viscid. The salt taste of the expectoration in the early stage of bronchitis, denotes the increase of saline matter in it. The presence of the chlorides in the substance of an inflamed lung, has been shown to correspond with their absence from the urine in pneumonia. Thus the inflamed vessels seem to acquire for the time being new excretory action. In addition to the above, inflammatory effusions generally contain the solids natural to the part, such as mucous globules, epithelium scales, epidermis and also blood corpuscles. For want of sufficient time, I am obliged to pass on to the consideration of the results or events of inflammation. Inflammation may result in resolution, effusion, suppuration, or gangrene. Did my time permit, I would be glad to go into the details incident to these different results; but I can only discuss one of them, namely—suppuration. This term signifies the formation of pus as a product of inflammation. Pus is an opaque, greenish or yellowish-white liquid, of creamy consistence, and comparatively odorless. It is chemically composed of water, duetoxide of protein forming the cell walls, tritoxide of protein and albumen in solution, fat, extractive matter, and the same salts as those in the blood. The circumstances which determine suppuration as a result of inflammation, are chiefly a certain intensity and duration of inflammation, the access of air to the part, or a peculiar condition of the blood. Intensity and continuance of inflammation comprise the persistence of the two chief elements in the process, determination of blood and obstruction. The access of air to a wound, or to a serous membrane, is well known to promote the formation of pus.

A limited access of air to a large quantity of pus, leads to a decomposition of the matter, and the production of sulphurretted hydrogen, which acts as a deleterious poison on living structures. That a peculiar condition of the blood promotes the occurrence of suppuration after inflammation is obvious, from the readiness with which all wounds, scratches and pimples then fester, and with which inflammation of no peculiar intensity, leads to the early formation of pus in different structures. In most cases the process of suppuration is limited by solid effusion, which may be the remains of the earlier product of the inflammation, or it may be thrown out expressly for the purpose of defending the adjoining structure from the operation of the pus, evidently so noxious a matter. A collection of pus thus circumscribed is called an *abscess*. The treatment of *abscess* is what I wish to call your particular attention to. Not because you have not had this subject thoroughly ventilated by lectures and clinical demonstrations, but because of the difficulty you will probably encounter in treating abscess, and also, because to be familiar with treatment in early practice, is advantageous. After an abscess has been opened, it will continue to discharge pus for an indefinite time, depending upon a variety of conditions, some of which will be under your control. In a healthy subject, a process of healing takes place by an increased effusion of lymph, throughout the interior of the abscess, and the growth of new vessels in this lymph in the form of granulations. Pus is still formed, by the degeneration of the superficial layer of exudation corpuscles, and a free vent must be given this pus, until the growth of the granulations, and the contraction of the walls, shall have obliterated the cavity of the abscess. To produce this return to an approximation to healthy conditions, is always desirable, and such a result is generally attainable, when *proper* remedies *are* used, and *improper* remedies are *not* used.

Abscesses havs been treated by our profession in a routine manner, regardless of the extent of either hard or soft tissue involved, and I regret to say, with not very flattering results, if we are to judge by the society reports and by the list of inquiries on the subject of their successful treatment in the dental journals. Creosote or carbolic acid, are the two remedies universally recommended, and, I may properly add, universally used, for the cure of abscess in all stages and conditions, that is, whether of recent origin or when a chronic diseased condition exists, and I wish to state right here that in neither condition is either creosote or carbolic acid generally indicated, nor should either be generally used. In all cases of abscess where there is a discharge of pus through the process and gum, and where the sac can be reached through this channel, immediately fill the root of the tooth, thereby closing that channel, and leaving all disease outside the tooth. From this course I never deviate. Should a probe through the gum reveal rough edges of bone, the best way is to remove these edges with burs and chisels. This plan facilitates the more rapid return to normal conditions, and renders it, I believe, more sure than the use of any medicine, (which would tend to produce exfoliation of the dead bone,) and at the same time has a tendency to leave the soft tissue in a favorable condition for healing. In short, direct your operations toward the removal of the cause, and nature will generally effect a cure. I cannot illustrate my position on this subject more clearly, perhaps, than by calling your attention to three cases:

Case I. Mr. D., of lymphatic temperament, called with an abscess at apex of root of right superior lateral incisor. The tooth had been filled with gold on lingual surface by a neighboring dentist, and had remained comfortable a number of months. It was so loose that I could have removed it with my fingers, and very sore to the touch. I opened the abscess freely, which operation soon relieved the patient of pain. I then removed the filling and opened into the nerve canal through the lingual surface of the tooth. Now, gentlemen, here is a case which, in the hands of the average practitioner, would have been dosed with creosote, both through the tooth and through the gum, although of a recent origin, and in a healthy patient, for, as I have already said, this is the usual way. I washed out the nerve canal with water, made a rope of soft gold foil, and immediately filled the nerve canal, nearly to the original position of the pulp proper, or that portion which occupied the center of the crown of the tooth, and dismissed my patient until the next morning, at which time I completed the filling and discharged the patient except with the request that he notify me if any trouble should occur. I saw him the fourth day after finishing the filling, and found the case perfectly cured, and cured by my not retarding the process by the use of creosote or carbolic acid. To have used either of these remedies would have prevented the case from healing so promptly. So much for a recent abscess.

Case II. Miss C., nervous temperament, called, having a right inferior first molar filled with amalgam, with the filling leaking, and a chronic discharge through the gum of six years standing. I removed the filling and found the roots had never been more than half filled, hence the abscess and chronic discharge. I removed all the debris from the roots, and, without further treatment, filled the roots with oxychloride of zinc, and the crown with gold, the operation lasting three hours, and dismissed the patient for a week, at which time I found the discharge much decreased, and in three weeks the cure was perfect. I saw the case within a month and found that no discharge had existed since dismissing the patient, three years ago. This case being connected with a lower molar, was one which is generally regarded as being most difficult to cure by any method of treatment. By removing thoroughly the cause it was cured without treatment.

Case III. This was a case of oral surgery. The cause of the disease was unknown to the patient. The discharge was through the cheek, on a line with the roots of the first left inferior molar. This abscess and discharge had existed four years, and had been treated with creosote and carbolic acid, and had been probed, and injected, and poulticed, but still the discharge remained. I mention these facts that you may know how much of the orthodox treatment an abscess will endure, and not be affected by it, except to grow worse, and also how much a patient will endure, and not be influenced, except to break a commandment with which I infer you are all familiar. This case was pronounced incurable. In treating it I operated by removing the soft parts diseased, until I came in contact with the external wall of the alveoli, which I found diseased. With chisels, and the use of large burs in my dental engine, I removed thoroughly all rough edges of bone, and readily determined when I had reached living bone, not only by the peculiar touch, but also by my patient again producing a compound fracture of the commandment already mentioned. I then carefully washed the parts with a very weak solution of chloride of zinc, brought the edges of soft tissue together, and held them there by two wire sutures, and covered the wound with adhesive plaster, as additional support to the sutures, and to exclude the air, the effect of which I have already mentioned. No other treatment was used, either local or general, and a perfect cure was secured. The operation was performed last January, ten months since, and the parts remain in perfect health, with scarcely any scar perceptible. Gentlemen, I am clearly of the opinion that had I used any of the remedies usually employed in such cases, I should not have secured such prompt and favorable results, and would have left a more perceptible scar, which would have been due to the protracted process of healing caused by my using any local treatment. Other cases might be mentioned, but these three are sufficient for my purpose, as they include both acute and chronic abscesses, in which both ossific and soft tissues were involved. Now, gentlemen, I do not presume to say that these cases might not have been cured by the use of such local remedies as are indicated in the books, but I do presume to say that none of the three cases, herein described, could have been cured by any additional medication, as quickly as they were cured, nor as pleasantly for the patient, which considerations merit the attention of any one interested.

I wish to impress you with three points in treating abscesses: First, remove if possible the cause; second, do not medicate unless you are sure such a course is clearly indicated; third, when you are satisfied a medicament is necessary, be careful that you use the one indicated. For instance, take a case of simple abscess without necrosis. In such a case, creosote is admissible, and may be sometimes advantageously used, to a *limited extent.* (I doubt whether more than one case in ten, as presented to the dentist for treatment, will involve any necrosis.) In the case just cited, the antiseptic influence of creosote is indicated, if *any* medicament is. But in a case where necrosis *does* exist, the antiseptic influence would render creosote objectionable, because it would tend to *prevent* the process of exfoliation which must either take place, or the dead bone must be removed by instruments, before a permanent cure can be produced. If this is not accomplished, the re-establishment of the disease is liable to ensue at any time.

Dr. J. N. Farrar, of Brooklyn, has been making a series of experiments, testing the relative merits, of the various preparations of sulphuric acid and creosote. He sums up the following conclusions:

"First, that the chemical activity of an aqueous solution of sulphuric acid upon dead bone, and teeth, is far greater than that of an alcoholic solution.

"Second, that simple abscess unaccompanied by necrosis, which is by far the larger class, indicates the antiseptic treatment, as it will cure the abscess, and tend to preserve the dead root of the tooth involved.

"Third, that necrosis without teeth being involved, does not call for the antiseptic treatment, because it would tend to prevent decomposition of the necrosed bone; but does indicate an aqueous solution of sulphuric acid, which may or may not include certain other stimulants.

"Fourth, that necrosis, with teeth involved, does not call for either antiseptic agents, or the aqueous solution of sulphuric acid, as the former will tend to prevent decomposition of the dead ossific tissue, desired to be disposed of; and the latter, if it can be used in sufficient quantities to promote decomposition of bone, will also chemically act upon and injure the dead root of the tooth involved. Such cases indicate a solution like the aromatic sulphuric acid, for while it will stimulate the surrounding living tissue to a healthier condition, and permit, and perhaps, in a very trifling degree, hasten the natural process of decomposition of porous necrosed bone, it will not act upon the tooth sufficiently to injure it."

The classification of treatment just quoted, indicates the following classes of abscess: First, abscess unaccompanied with necrosis; second, necrosis without teeth involved; third, necrosis with teeth involved. Thus you see, gentlemen, that there are three distinct conditions, clearly indicating a different remedy for the cure of each. You will also readily observe, that to use creosote in the second or third class, would retard the desired results, and to use the aqueous solution of sulphuric acid in the third class would tend to produce decomposition of the root of the tooth, as well as to produce a removal of the dead bone, and that in this class, the aromatic solution of sulphuric acid is indicated, just where the aqueous solution of sulphuric acid and creosote are not indicated. In determining what remedy is indicated, in order to most successfully treat these cases, you will have to use your personal judgment. Experience will teach you to determine what medication is necessary, if you profit by it, and you are to be congratulated, if you are, at the same time taught what *not* to use. We have a sufficient number of remedies to meet all these conditions, but what remedies to use, and where, and how, to use them, is the most difficult problem—especially for the young practitioner to solve. In forming a diagnosis, you will find that a knowledge of the mechanism of organs in health and disease, and of the physical laws to which that mechanism is subjected, is the best aid to the study of physical signs. And an accurate acquaintance with the structure and functions of healthy and diseased tissue, and with the vital laws which influence them, is the best guide to the comprehension of vital symptoms. These symptoms often obscure, but a determination upon your part to excel as diagnosticians, will surely win for you the ability to overcome all difficulties in this direction. All classes of indications ought to be carefully taken into account, in forming a diagnosis, and the more fully the physical and vital properties which constitute them are understood, the more available will signs and symptoms be in leading to correct conclusions, both in diagnosis and treatment.

Gentlemen, I have endeavored, so far as I have been able to discuss this subject in a single essay, to at least give you a start in the investigation of the subject of inflammation, abscess and necrosis, and the treatment indicated, or perhaps I should say, the treatment not indicated. For I think it is a fact that young practitioners are liable to overtreat, if I may use the term, in their anxiety to relieve their patients. Although this anxiety is commendable, it is perhaps well to be advised of this tendency, before committing the error, and so avoid that which those who have preceded you, have only learned to avoid by experience. Which experience may have been a good thing for the dentist, but not so profitable to the patient.

In conclusion, gentlemen, I desire to remind you, that your opportunities for acquiring a thorough knowledge of your profession, have never been so good as they are now, and an appreciation of this fact will enable you to become honorable members of a profession, which has advanced more rapidly than any other ever has done, and by your aid, will, I trust, continue to advance in the future, as it has in the past.—*Dental Register.*

---

